# Theoretical insights into interfacial stability and ionic transport of Li_2_OHBr solid electrolyte for all-solid-state batteries†

**DOI:** 10.1039/d2ra06921k

**Published:** 2022-12-02

**Authors:** Bo Liu, Piguang Liao, Xiaowen Shi, Yufeng Wen, Qingdong Gou, Meidong Yu, Shenlin Zhou, Xinyuan Sun

**Affiliations:** College of Mathematics and Physics, Jinggangshan University Ji'an Jiangxi 343009 China liubo@jgsu.edu.cn; Science and Technology Innovation Development Center Ji'an Jiangxi 343006 China

## Abstract

Li-rich antiperovskite materials are promising candidates as inorganic solid electrolytes (ISEs) for all-solid-state Li-ion batteries (ASSLIBs). However, the material faces several pressing issues for its application, concerning the phase stability and electrochemical stability of the synthesized material and the Li-ion transport mechanism in it. Herein, we performed first-principles computational studies on the phase stability, interfacial stability, defect chemistry, and electronic/ionic transport properties of Li_2_OHBr material. The calculation results show that the Li_2_OHBr is thermodynamically metastable at 0 K and can be synthesized experimentally. This material exhibits a wider intrinsic electrochemical stability window (0.80–3.15 V) compared with sulfide solid electrolytes. Moreover, the Li_2_OHBr displays significant chemical stability when in contact with typical cathode materials (LiCoO_2_, LiMn_2_O_4_, LiFePO_4_) and moisture. The dominant defects of Li_2_OHBr are predicted to be V_Li^−^_ and Li_i_^+^, corresponding to lower Li-ion migration barriers of 0.38 and 0.49 eV, respectively, while the replacement of some of the OH^−^ by F^−^ is shown to be effective in decreasing migration barriers in Li_2_OHBr. These findings provide a theoretical framework for further designing high performance ISEs.

## Abstract 2

Li-rich antiperovskite materials are promising candidates as inorganic solid electrolytes (ISEs) for all-solid-state Li-ion batteries (ASSLIBs).

## Graphical abstract

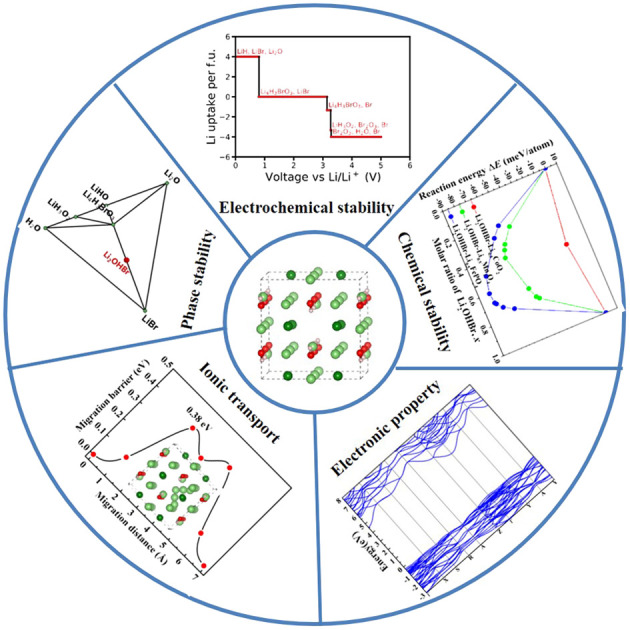

## 1. Introduction

In recent years, inorganic solid electrolytes (ISEs) have received wide attention to replace organic liquid electrolytes currently used in commercial lithium-ion batteries.^1^ Unlike organic liquid electrolytes, ISEs are nonflammable, nonvolatile, and have no liquid leakage problem, and are also expected to overcome the phenomenon of lithium dendrites, thus they have high safety performance.^2^ Moreover, ISEs have the potential to improve interfacial stability, which could enable the application of a high-voltage cathode and even lithium metal anode.^3^ In terms of Li-ion transport properties, several ISEs have been reported, such as the Li_10_GeP_2_S_12_,^4^ Li_7_P_3_S_11_,^5^ and lithium-rich anti-perovskites (LRAP),^6^ with high Li^+^ conductivities comparable to or even surpassing those of traditional liquid electrolytes.

One promising class of ISEs is antiperovskites Li_3−*n*_OH_*n*_X (*n* = 0–1, X = Cl, Br) for ASSLIBs.^7^ For example, Zhao *et al.* first experimental reported that Li_3_OCl and Li_3_O(Cl_0.5_Br_0.5_) showed a high ion-conductivity (>10^−3^ S cm^−1^ at 300 K) with low activation energies (0.18–0.26 eV).^8^ Subsequently, a good stability and low Li^+^ vacancy migration barrier of Li_3_OX (X = Cl, Br) was verified by first-principles calculations.^9^ Sugumar *et al.* reported the successful preparation of Li_2_OHBr by dry ball-milling of LiOH and LiBr at room temperature, which obtained high ion conductivity of 1.1 × 10^−6^ S cm^−1^ with the activation energy of 0.54 eV.^10^ Recently, Yamamoto *et al.*^11^ reported that an ASSLIB composed of Li/Li_2_OHBr/Fe_2_(MoO_4_)_3_ were fabricated by pressing at room temperature, which exhibited good charge–discharge performance and excellent cycle stability. Zhao and co-workers^12^ proposed the Li_2_OHBr as a protective layer for the Li_1.5_Al_0.5_Ge_1.5_(PO_4_)_3_ (LAGP) solid electrolyte to prevent the side reaction caused by direct contact between LAGP and Li metal anode. For practical applications, whether Li_2_OHBr material acts as a solid electrolyte or protective layer, it is crucial that the material shows good thermodynamic stability, electrochemical stability and fast Li-ion diffusion, which are the keys to ameliorating the electrochemical and rate performance of Li_2_OHBr material. Moreover, Li_2_OHBr should possess the ability of moisture resistance and oxidation resistance, which will simplify the packaging of ASSLIBs in practice. However, in-depth understanding of these important issues, has been hindered by the complicated synthesis and measurement conditions during the experiments. Therefore, it is critical that we explore the fundamental issues of the phase stability, electrochemical stability, chemical stability and electron/ion transport mechanism of Li_2_OHBr through reliable theoretical approaches to elucidating the main behind physics mechanism.

In this work, we employ first-principles calculations to assess the phase stability, interfacial stability against electrode material, defect chemistry and electron/ion transport mechanism of anti-perovskites Li_2_OHBr. We predict a wide electrochemical window and low chemical reactivity for Li_2_OHBr, ensuring that this material is thermodynamically stable under high-voltage operation and in air. The analysis Li-ion transport mechanism shows the existence of low migration barriers involving charge carriers (V_Li^−^_, Li_i_^+^) in Li_2_OHBr. We also study the effect of F^−^ substitution of OH^−^ on the Li^+^ migration barriers in Li_2_OHBr. The computational approach in this work can be extended to the design of other ISEs system.

## 2. Computational methods

All calculations are performed based on density functional theory (DFT) by using the projector augmented wave method, as implemented in the Vienna *ab initio* Simulation Package (VASP).^13^ The generalized gradient approximation (GGA) with Perdew–Burke–Ernzerhof (PBE) is applied to treat the electronic exchange–correlation interactions.^14^ The cutoff energy is set to 520 eV. The atomic force and energy convergence parameters are consistent with Materials Project (MP)^15^ for all calculations. Based on DFT ground-state energies in the MP database, the phase stability and interfacial stability (including electrochemical and chemical stability) of Li_2_OHBr are evaluated using the same scheme in the previous work.^16^

The phase stability of Li_2_OHBr is assessed by computing the energy above convex hull, corresponding the decomposition energy to the thermodynamic phase equilibria. The electrochemical window of Li_2_OHBr is calculated by using the Li grand potential phase diagram. In this method, the grand potential *ϕ* of the Li_2_OHBr is defined as:^16^

1 *ϕ*[*c*, *μ*_Li_] = *E*[*c*] − *n*_Li_[*c*]*μ*_Li,_

where *μ*_Li_, *n*_Li_[*c*], and *E*[*c*] are the Li chemical potential, Li concentration and DFT energy of composition *c*, respectively.

The chemical stability of Li_2_OHBr/cathode interfaces are determined by estimating the reaction between the Li_2_OHBr and cathode with the lowest reaction energy (Δ*E*),^17^ namely:2
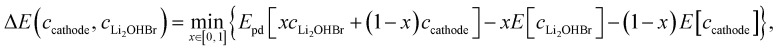
where *x* is the mixing fraction of the cathode/coating compositions, *E*[*c*_cathode_] and *E*[*c*_Li_2_OHBr_] are DFT energies of the cathode and Li_2_OHBr, respectively. *E*_pd_[*c*] represents the DFT energy corresponding to the given composition *C* in the chemical reaction equilibrium.

The defect formation energy *E*_f_(*i*, *q*) with a defect *i* at charge state *q* is calculated *via* the following equation:^18^

3 *E*_f_(*i*, *q*) = *E*_tot_(*i*, *q*) − *E*_tot_(Li_2_OHBr, bulk) − *nμ*_*i*_ + *q*(*ε*_F_ + *E*_V_),

where *E*_tot_(Li_2_OHBr, bulk) and *E*_tot_(*i*, *q*) are the DFT energies of Li_2_OHBr supercell without and with a defect, respectively. *n* is the number of Li or Li_2_O compound added (*n* > 0) or removed (*n* < 0) from the perfect supercell, and *μ*_*i*_ is the chemical potential of Li (bcc structure) or Li_2_O (fcc structure) compound. The Fermi level (*ε*_F_) is defined relative to the valence-band maximum (*E*_V_) of perfect Li_2_OHBr and varies with defect concentration.

## 3. Results and discussion

### 3.1 Structural and electronic property

**Fig. 1:**
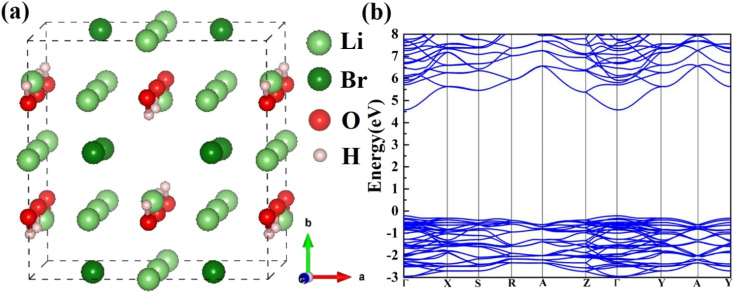
(a) Atomic structure and (b) electronic band structure of Li_2_OHBr. The ball colors green, dark red, red, and gray indicate Li, Br, O, and H sites, respectively.

The Li_2_OHBr orthorhombic structure with space group *Cmcm* is constructed, as shown in Fig. 1(a). Table 1 lists the optimized lattice parameters of Li_2_OHBr obtained by DFT calculations, together with the data from Howard *et al.*^19^ The lattice parameters are *a* = 8.015 Å, *b* = 8.152 Å, and *c* = 7.944 Å, which are agree with the experiment data (within 2% error). To obtain the electronic properties of Li_2_OHBr, the electronic band structure of Li_2_OHBr is evaluated. Since the GGA-PBE functional generally underestimate the band gap, the advanced SCAN meta-GGA functional is used to provide a rigorous result.^20^Fig. 1(b) shows that the direct band gap of Li_2_OHBr is 4.79 eV, and its VBM and CBM are both located at the *Γ* point of the first Brillouin zone. The large band gap indicates that Li_2_OHBr is electron insulator, which can effectively block electron leakage and prevent electrode corrosion. The band gap between the valence band maximum (VBM) and the conduction band minimum (CBM) provides an upper limit for the electrochemical window of solid electrolytes. When the chemical potential of the electrode/solid electrolyte is mismatched, *i.e.*, thermodynamically unstable, a chemical reaction between the two materials will occur spontaneously upon contact.

**Table 1:** Relaxed lattice parameters and atomic coordinates of Li_2_OHBr in the orthorhombic

System		Calc. (this work)			Exp. (ref. 19)		
Lattice parameters		*a* (Å)	*b* (Å)	*c* (Å)	*a* (Å)	*b* (Å)	*c* (Å)
		8.015	8.152	7.944	8.010	8.030	7.880
		*α* (°)	*β* (°)	*γ* (°)	*α* (°)	*β* (°)	*γ* (°)
		90	90	90	90	90	90
Atom	Wyckoff	*x*	*y*	*z*	*x*	*y*	*z*
O	8f	0.000	0.744	0.514	0.000	0.741	0.513
H	8f	0.000	0.823	0.414	0.000	0.824	0.419
Cl	8g	0.749	0.489	0.250	0.747	0.487	0.250
Li	8d	0.250	0.250	0.000	0.250	0.250	0.000
Li	4b	0.000	0.500	0.000	0.000	0.500	0.000
Li	4c	0.000	0.195	0.250	0.000	0.197	0.250

### 3.2 Phase stability and interfacial stability

**Fig. 2:**
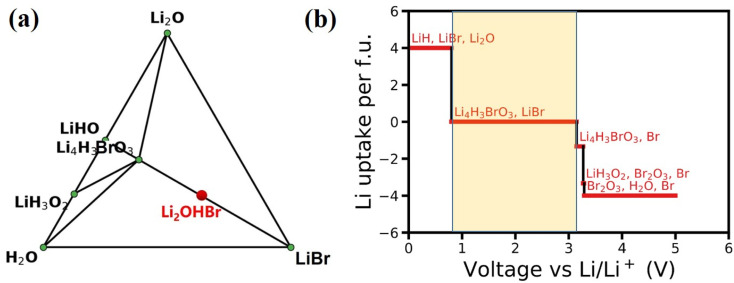
(a) Li–H–O–Br quaternary phase diagram. The metastable Li_2_OHBr is marked in red font. (b) The voltage profiles and phase equilibria of Li_2_OHBr at different potentials. The light yellow region indicates the electrochemical stability window range.

The feasibility and complexity level of the experimental synthesis of one given material could be evaluated by its phase stability. In general, for a particular component that does not have a stable phase, the corresponding system will decompose into a stable phase around its component coordinate points. The Li–H–O–Br quaternary phase diagram is constructed at 0 K by minimizing the formation energies of various compositions, as shown in Fig. 2(a). The phase diagram indicates that Li_2_OHBr is energetically unstable duo to positive formation energy with respect to that form the Li_4_H_3_BrO_3_ and LiBr. However, the energy above hull (*E*_hull_) of Li_2_OHBr is only 11.6 meV per atom, suggesting that Li_2_OHBr is likely to be metastable at 0 K against possible decomposition products, and therefore may be stabilized by external conditions (such as pressure, temperature, and entropy).^10^ For example, the Li_2_OHBr was synthesized from LiOH and LiBr starting materials by sintering method above 300 °C.^21^ In fact, successful synthesis of metastable phases has been widely reported for ISEs, such as Li_6_PS_5_Cl (21 meV per atom),^22^ Li_10_GeP_2_S_12_ (25 meV per atom)^4^ and Li_7_P_3_S_11_ (27 meV per atom).^23^

For the practical application of ASSLIBs, the ISEs should satisfy the conditions of good interfacial stability, including electrochemical stability and chemical stability.^24^ Using eqn (1), the phase equilibrium of Li_2_OHBr for a series of lithiation/delithiation reactions is predicted to obtain the electrochemical stability window. The detailed lithiation/delithiation reactions with *μ*_Li_ are listed in Table S1.† As shown in Fig. 2(b), Li_2_OHBr is oxidized to form Li_4_H_3_BrO_3_ and Br when the oxidation voltage is higher than 3.15 V. Meanwhile, Li_2_OHBr is reductively decomposed into LiH, LiBr and Li_2_O starting from 0.80 V. The calculated electrochemical stability window range of Li_2_OHBr are 0.80–3.15 V *vs.* Li/Li^+^. Table 2 shows that Li_2_OHBr has much wider electrochemical window than that of reported sulfides and oxides solid electrolyte, such as Li_10_GeP_2_S_12_ (1.71–2.14 V), Li_7_P_3_S_11_ (2.28–2.31 V), Li_6_PS_5_Cl (1.71–2.01 V), Li_2_PO_2_N (0.68–2.63 V) and Li_7_La_3_Zr_2_O_12_ (0.05–2.91).^25^ However, it should be pointed out that the dissociation of ISEs depends on kinetic factors, suggesting that the dissociation of the phases may be slowed down or interrupted under certain circumstances, such as slow electron/ion transport in dissociated phases. The above calculations assume complete thermodynamic equilibrium and no kinetic constraints in the reactions. Therefore, ISEs are expected to withstand a wider range of voltages in use than calculated, as measured by cyclic voltammetry (CV) using Li/Li_2_OHBr/Au cell, the electrochemical potential window of Li_2_OHBr was 1.7–3.5 V.^11^

**Table 2:** Summary of electrochemical window (EW), equilibria phases at reduction and oxidation potentials of some typical solid-state electrolytes

Solid-state electrolytes	EW *vs.* Li/Na (V)	Equilibria phase at reduction potential	Equilibria phase at oxidation potential
Li_2_OHBr (this work)	0.80–3.15	LiH, LiBr, Li_2_O	Li_4_H_3_BrO_3_, Br
Li_2_OHCl (ref. 26)	0.82–3.15	LiH, LiCl, Li_2_O	LiH_2_ClO_5_, H_2_O, LiCl
Li_3_OCl (ref. 9)	0–2.55	Li_3_OCl	Li_2_O_2_, LiCl
Na_3_OBr (ref. 27)	0–1.79	Na_3_OBr	Na_2_O_2_, NaBr
Li_10_GeP_2_S_12_ (ref. 25)	1.71–2.14	Li_4_GeS_4_, Li_2_S, P	Li_3_PS_4_, GeS_2_, S
Li_3_PS_4_ (ref. 25)	1.71–2.31	Li_2_S, P	P_2_S_5_, S
Na_3_PS_4_ (ref. 27)	1.39–2.45	Na_3_P, Na_2_S	P_2_S_5_, S
Li_7_P_3_S_11_ (ref. 25)	2.28–2.31	Li_3_PS_4_, P_4_S_9_	P_2_S_5_, S
Li_6_PS_5_Cl (ref. 25)	1.71–2.01	Li_2_S, LiCl, P	Li_3_PS_4_, LiCl, S
Li_2_PO_2_N (ref. 25)	0.68–2.63	Li_3_P, LiPN_2_, Li_2_O	P_3_N_5_, Li_4_P_2_O_7_, N_2_
Li_7_La_3_Zr_2_O_12_ (ref. 25)	0.05–2.91	Zr_3_O, La_2_O_3_, Li_2_O	Li_2_O_2_, La_2_O_3_, Li_6_Zr_2_O_7_

**Fig. 3:**
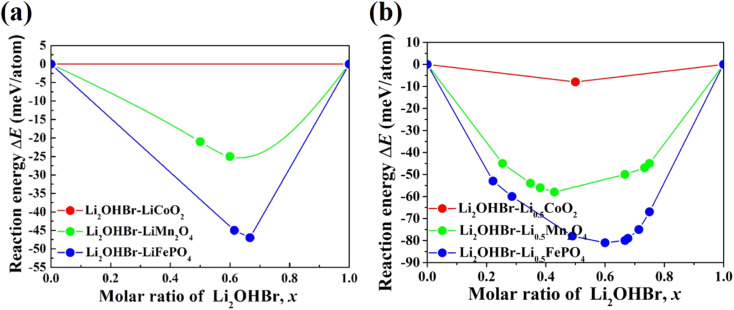
Predicted reaction energies Δ*E* between Li_2_OHBr and various cathode materials in both (a) fully-discharged and (b) half-charged states. The reaction energy Δ*E*_min_ corresponds to the lowest mutual reaction energy at a ratio of *x*.

The chemical stability between the Li_2_OHBr and various cathodes is calculated by using eqn (2). Three typical cathode materials (*e.g.*, layered LiCoO_2_, spinel LiMn_2_O_4_, olivine LiFePO_4_) are considered for fully-discharged and half-charged state. Fig. 3 shows the predicted reaction energies between the Li_2_OHBr and cathode materials, and the corresponding reaction products are listed in Table S2.† The mutual reaction energy Δ*E*_min_ of Li_2_OHBr with both fully-discharged cathodes are predicted to have low reaction energies (0 < |Δ*E*_min_| < 50 meV per atom) in Fig. 3(a). The chemical reactivity sequence for a fully-discharged cathodes with the Li_2_OHBr is LiFePO_4_ > LiMn_2_O_4_ > LiCoO_2_, suggesting that LiCoO_2_ cathode seems to have better interfacial compatibility with the Li_2_OHBr. An increased chemical reactivity of Li_2_OHBr with half-charged cathodes are observed from fully-discharged to half-charged cathode states, as shown in Fig. 3(b). It is worth noting that the Li_2_OHBr has either no reaction or a negligible driving force against LiCoO_2_ cathode, showing a significant thermodynamic chemical stability. Certainly, for all interfacial reactions Δ*E*_min_ < 0, a thermodynamically unstable interface is formed when Li_2_OHBr is in contact with high-voltage cathodes, which may result in the formation of unwanted interfacial byproducts, thereby reducing the rate capacity and electrochemical performance of ASSLIBs. Therefore, further experiment techniques are awaited to assess potential interfacial products, such as X-ray photoelectron spectroscopy (XPS), X-ray absorption spectroscopy (XAS), and electron microscopy (EM).^28^

The air stability of ISEs is another issue involved in electrolyte handling and battery assembly in the development of ASSLIBs, where the electrolyte will inevitably be exposed to air and undergo structural changes if it is not chemically stable.^29^ For example, using the first-principles calculations, Zhang *et al.*^30^ revealed the thermodynamic and kinetic mechanism in the reaction of Li_10_GeP_2_S_12_ with H_2_O in air to produce H_2_S gas. Here, the stability of Li_2_OHBr toward air is also studied using reaction energies Δ*E* calculation for the reaction with moisture, the higher negative value indicates the material is strongly favorable to react with moisture. The estimation of driving force for Li_2_OHBr when exposed to air *via* following reaction:

4 3Li_2_OHBr + 2H_2_O → 2LiOH_2_Br + Li_4_(OH)_3_Br,

5 2Li_4_(OH)_3_Br + 3CO_2_ → 2LiOH_2_Br + 3Li_2_CO_3_ + H_2_O.

The estimated value of Δ*E* is only −1 meV per atom when Li_2_OHBr reacts with H_2_O. While Li_4_(OH)_3_Br forms as a hydrolysis intermediate and subsequently reacts favorably with CO_2_ to produce LiOH_2_Br, Li_2_CO_3_ and H_2_O (Δ*E* = −118 meV per atom). Therefore, it is suggested that the Li_2_OHBr solid electrolyte is stable in dry air. To understand the degradation mechanism of ISEs exposed to air, some experimental techniques, such as *in situ* scanning/transmission electron microscopy, neutron ray diffraction depth analysis and synchrotron X-ray imaging technologies, have been performed to track local nanoscale chemical evolution and structural information of interfacial phases.^31^

### 3.3 Ionic transport mechanism

#### 3.3.1. Defect structure and formation energy

Defects in ISEs have a remarkable effect on Li-ion diffusion, and the types of defects are determined by synthesis conditions (*e.g.*, chemical potential) affecting their formation energies.^32^ To elucidate the ion transport mechanism, it is necessary to understand which defects favor the diffusion of lithium ions in Li_2_OHBr. Herein, four defect types in Li_2_OHBr are considered, including lithium vacancy 
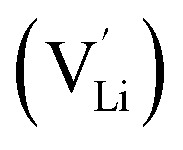
, lithium interstitial 
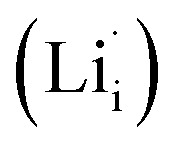
, lithium Frenkel defect pair 
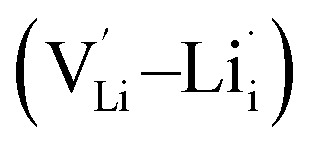
 and Li_2_O Schottky defect pair 
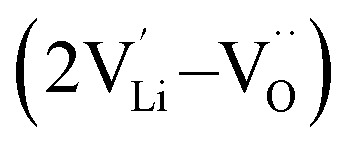
, as per our previous work.^33^ Among these defects, lithium Frenkel defect and Li_2_O Schottky defect pair in Li_2_OHBr are described as follows:

Lithium Frenkel defect:6
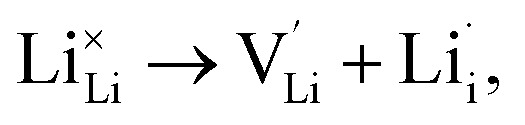


Li_2_O Schottky defect:7
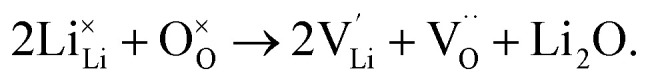


**Fig. 4:**
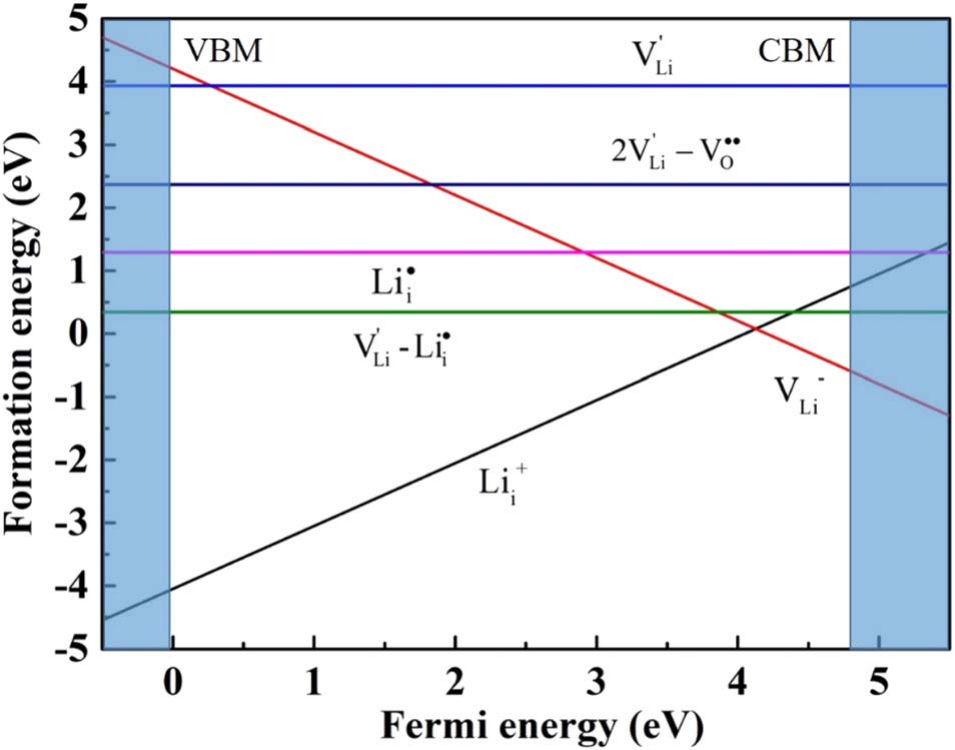
Formation energies of lithium vacancy and interstitial at different charge states.

According to the symmetry of Li_2_OHBr, the possible defect configurations and formation energies are investigated to obtain the lowest energy configuration. The possible defect configurations include two different lithium vacancy defects 
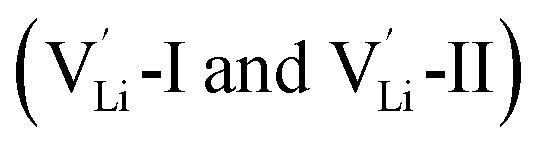
, one lithium interstitial defect 
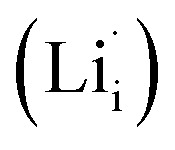
, two lithium Frenkel defect pair (V_near_ and V_far_), and three Li_2_O Schottky defect pair (V_adjacent_, V_separated-1_, and V_separated-2_) in the ESI of Fig. S1 and S2.† The formation energies of four defect types 
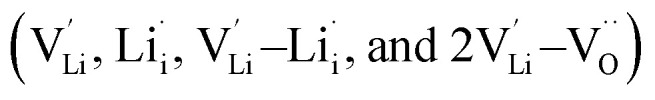
 are calculated by eqn (3) in the neutral state, as listed in Table 3. By comparing the formation energies, it is found that the dominant defect configuration is V_near_, and the corresponding defect formation energies is 0.34 eV. In contrast, single 
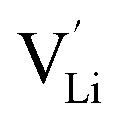
 and 
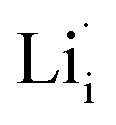
 show higher defect formation energies (3.93 eV and 1.29 eV), implying a lower concentration of lithium vacancy and lithium interstitial defect in neutral Li_2_OHBr. In addition, the defect formation energies of lithium vacancy and interstitial at different charge states *q* as a function of Fermi level are also calculated in Fig. 4. The results show that the formation energies of Li_i_^+^ and V_Li^−^_ are lower than those of 
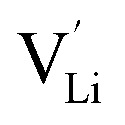
, 
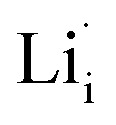
, 
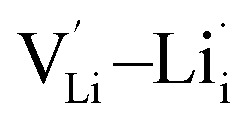
 and 
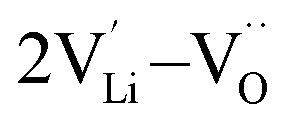
, suggesting that the Li_i_^+^ and V_Li^−^_ may be the main defect types in Li_2_OHBr at room temperature. Therefore, charged defects (Li_i_^+^ and V_Li^−^_) should act as charge carriers in Li_2_OHBr and will be used to calculate the migration barriers in the next section.

**Table 3:** Defect formation energies corresponding to various defect types and configurations in Li_2_OHBr

Defect type	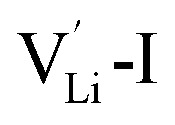	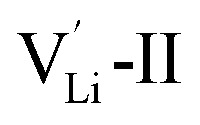	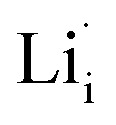	V_near_	V_far_	V_adjacent_	V_separated-1_	V_separated-2_
*u*_*i*_ (eV)	−1.89	−1.89	−1.89	—	—	−14.35	−14.35	−14.35
*E*_f_ (eV)	3.93	4.26	1.29	0.34	0.62	3.01	2.37	2.75

#### 3.3.2. Li-ion migration *via* vacancy and interstitial mechanism

**Fig. 5:**
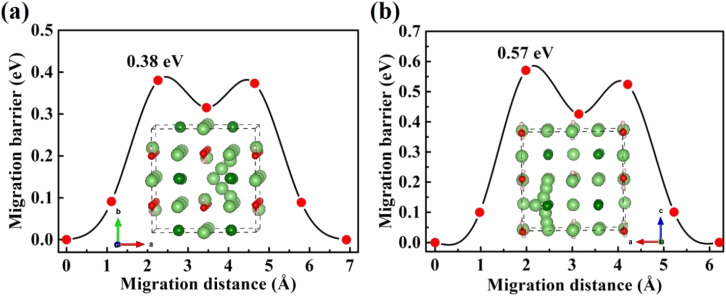
Diffusion pathway and migration barriers of V_Li^−^_ in Li_2_OHBr (a) along the *ab*-plane and (b) along the *c*-axis.

**Fig. 6:**
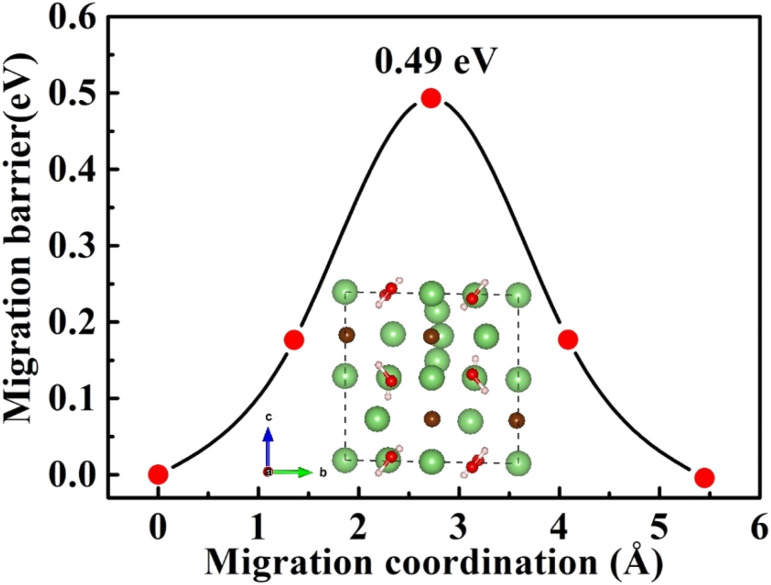
Diffusion pathway and migration barriers of Li_i_^+^ in Li_2_OHBr along the *c*-axis.

A high ionic conductivity at room temperature is one of most important indices for the practical application of ISEs in ASSBs. Herein, the Li-ion diffusion pathways and migration barriers of charge carriers (V_Li^−^_, Li_i_^+^) in Li_2_OHBr are studied using CI-NEB method. Fig. 5 shows the diffusion path of a single lithium vacancy (V_Li^−^_) along the *ab*-plane and *c*-axis in Li_2_OHBr, corresponding to the migration barriers are 0.38 and 0.57 eV, respectively. For the direct lithium interstitial (Li_i_^+^) migration in Li_2_OHBr, migration process from an interstitial site to its adjacent interstitial site needs to cross an energy barrier of 0.49 eV, as shown in Fig. 6. The energy barriers of V_Li^−^_ is slightly lower than that of Li_i_^+^, suggesting that V_Li^−^_ and Li_i_^+^ will be generated simultaneously when the migrating Li leaves the lattice site. Therefore, we can conclude that the charge carriers (V_Li^−^_ and Li_i_^+^) make main contribution to the ionic conductivity of Li_2_OHBr and that increasing the concentrations of V_Li^−^_ and Li_i_^+^ defects are crucial to obtain high Li-ion conductivity.

**Fig. 7:**
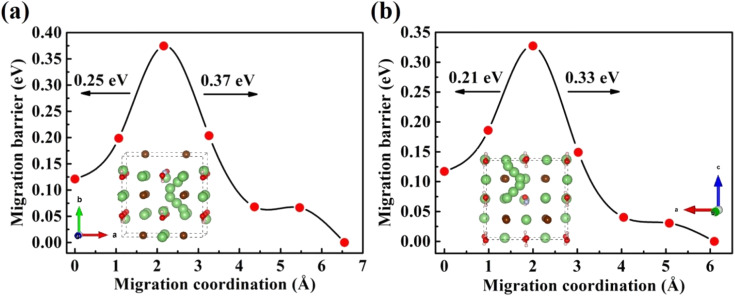
Diffusion pathway and migration barriers of V_Li^−^_ in F-doped Li_2_OHBr (a) along the *ab*-plane and (b) along the *c*-axis.

Furthermore, the doping of Li_2_OHBr with halogen element (F or Cl) may be one of the key factors to enhance the ionic conductivity. In Fig. 7(a) and (b), we present the lithium vacancy migration barriers obtained using CI-NEB calculations for F-doped Li_2_OHBr, namely Li_2_(OH)_0.875_F_0.125_Br. The results show that the migration barriers of Li_2_(OH)_0.875_F_0.125_Br is 0.37 and 0.33 eV along the *ab*-plane and *c*-axis, respectively, which is a lower value than that of pristine Li_2_OHBr. The main reason is that the substitution of F^−^ for OH^−^ increases the antiperovskite tolerance factor and favors a disordering of the OH^−^ orientation for Li_2_OHBr, as previous reported by Li *et al.*^21^ Therefore, the Li_2_OHBr can be doped by the substitution of OH^−^ by F^−^ is beneficial to reduce the migration barrier and improve the ionic conductivity.

## 4. Conclusions

In conclusion, the electronic properties, phase stability, interfacial stability, defect chemistry and Li-ions migration mechanisms of Li_2_OHBr have been systematically studied by the first-principles calculations. The calculations results indicate that Li_2_OHBr crystal structure is metastable by thermodynamics analysis. The electronic band structure shows that the Li_2_OHBr is an insulator with a wide direct band gap. The Li_2_OHBr has a wide electrochemical stability window that can be matched with the cathode materials. Moreover, the Li_2_OHBr also exhibits good chemical stability with typical cathode materials and in air. By comparing the defect formation energies in neutral and charged states, it is shown that charged Li_i_^+^ and V_Li^−^_ are the most dominant defect types in Li_2_OHBr. The Li_2_OHBr show the low migration barriers by using the CI-NEB calculation, while F-doped Li_2_OHBr has a lower migration barrier compared with pristine Li_2_OHBr. This work provides insights into the thermodynamic and kinetic process of Li_2_OHBr and demonstrates the potential of computational methods in the efficient design of future ISEs.

## Conflicts of interest

There are no conflicts to declare.

## Supplementary Material

RA-012-D2RA06921K-s001
