# Supplementary material for: Theoretical insights into interfacial stability and ionic transport of Li2OHBr solid electrolyte for all-solid-state batteries
Source: RSC Adv. 2022 Dec 2;12(53):34627–33. doi: 10.1039/d2ra06921k (PMC9716346; doi:10.1039/d2ra06921k)
Supplement: RA-012-D2RA06921K-s001 [file RA-012-D2RA06921K-s001.pdf]

Electronic Supplementary Material (ESI) for RSC Advances.
This journal is © The Royal Society of Chemistry 2022

Supporting Information

Theoretical insights into interfacial stability and ionic transport of Li_2OHBr solid electrolyte for all-solid-state batteries

Bo Liu,[a,b*] Piguang Liao,[a] Xiaowen Shi,[b] Yufeng Wen,[a] Qingdong Gou,[a] Meidong Yu,[a] Shenlin Zhou,[a] Xinyuan Sun[a]

[a] *College of Mathematics and Physics, Jinggangshan University, Ji'an, Jiangxi 343009, China*

[b] *Science and Technology Innovation Development Center, Ji'an, Jiangxi 343006, China*

*E-mail: Bo Liu (liubo@jgsu.edu.cn)

Table S1. Calculated plateaus for the lithiation and delithiation of Li_2OHBr solid-state electrolyte.

Potential ref. to Li/Li^+ (V)	μ_{Li} ref. to Li metal (eV)	Δn_{Li} per formula	Phase equilibria
0.80	-0.80	2.00	Li_2O, LiH, LiBr
		0	$Li_4H_3BrO_3$, LiBr
3.15	-3.15	-0.67	$Li_4H_3BrO_3$, Br

3.27	-3.27	-1.67	LiH_3O_2, Br_2O_3, Br
3.96	-3.29	-2.00	Br_2O_3, H_2O, Br

Table S2. Predicted reaction products and reaction energies (meV/atom) between cathodes ($LiCoO_2$, $LiMn_2O_4$ and $LiFePO_4$) and Li_2OHBr solid-state electrolyte.

Cathodes	Li_2OHBr, x	Phase equilibria	ΔE(meV/ atom)
$LiCoO_2$	-	$LiCoO_2$+Li_2OHBr	0
$Li_{0.5}CoO_2$	0.5	LiH_2BrO+$LiCoO_2$+LiBr+Li_2CoO_3	-8
$LiMn_2O_4$	0.60	LiH_2BrO+LiBr+Mn_3O_4+Li_2MnO_3	-19
	0.50	LiH_2BrO+LiBr+Mn_2O_3+Li_2MnO_3	-17
$Li_{0.5}Mn_2O_4$	0.75	$LiMnO_2$+LiBr+Li_2MnO_3+LiH_2BrO	-45
	0.733	Mn_3O_4+LiBr+Li_2MnO_3+LiH_2BrO	-47
	0.667	Li_2MnO_3+LiBr+$LiMn_2O_4$+LiH_2BrO	-50
	0.444	$LiMn_2O_4$+LiBr+$Li_5Mn_7O_{16}$+LiH_2BrO	-58
	0.381	$MnH_4Br_2O_2$+LiBr+$Li_5Mn_7O_{16}$+ LiH_2BrO	-56
	0.348	$MnH_4Br_2O_2$+LiBr+$Li_5Mn_7O_{16}$+Br	-54
	0.254	$MnH_4Br_2O_2$+Mn_2O_3+$Li_5Mn_7O_{16}$+Br	-45
$LiFePO_4$	0.667	$Fe(HO)_2$+LiBr+Li_3PO_4	-40
	0.615	$FeH_8Br_2O_4$+FeO+LiBr+Li_3PO_4	-39
$Li_{0.5}FePO_4$	0.75	FeO+$LiFeO_2$+LiH_2BrO+LiBr+Li_3PO_4	-67
	0.714	$FeHO_2$+FeO+LiH_2BrO+LiBr+Li_3PO_4	-75
	0.677	$FeH_8(BrO_2)_2$+FeO+$FeHO_2$+LiBr+Li_3PO_4	-79
	0.667	$FeH_8(BrO_2)_2$+Fe_2O_3+FeO+LiBr+Li_3PO_4	-80
	0.60	$FeH_8(BrO_2)_2$+Fe_2O_3+$LiFePO_4$+LiBr+Li_3PO_4	-81
	0.49	$FeH_8(BrO_2)_2$+Fe_2O_3+$LiFePO_4$+$LiFeBr_4$+Li_3PO_4	-78
	0.286	$LiFePHO_5$+$LiFePO_4$+$LiFeBr_4$+Li_3PO_4	-60
	0.222	$LiFePHO_5$+$LiFePO_4$+$LiFeBr_4$+$Li_3Fe_2(PO_4)_3$	-53

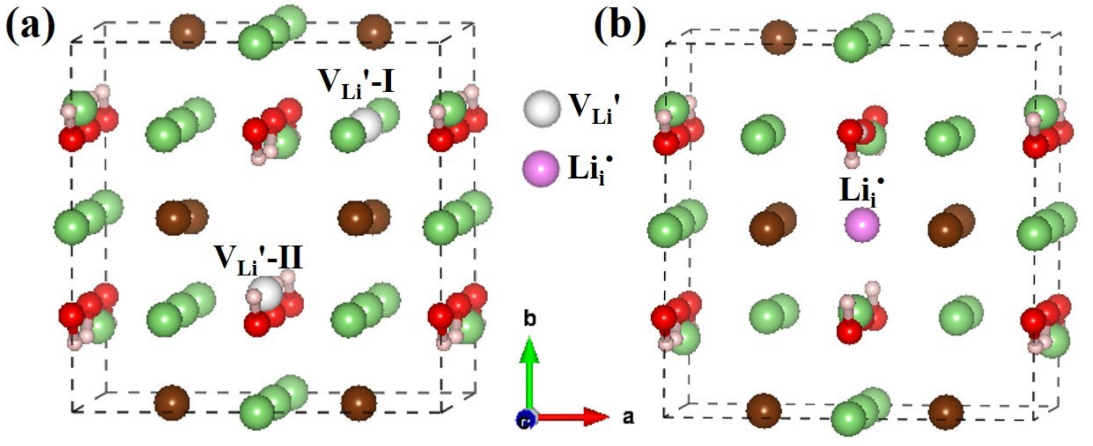

Figure S1. Possible defect configurations of the Li_2OHBr in (a) single lithium vacancy ($V_{Li}^{'}$) and (b) single lithium interstitial ($Li_i^{\bullet}$).

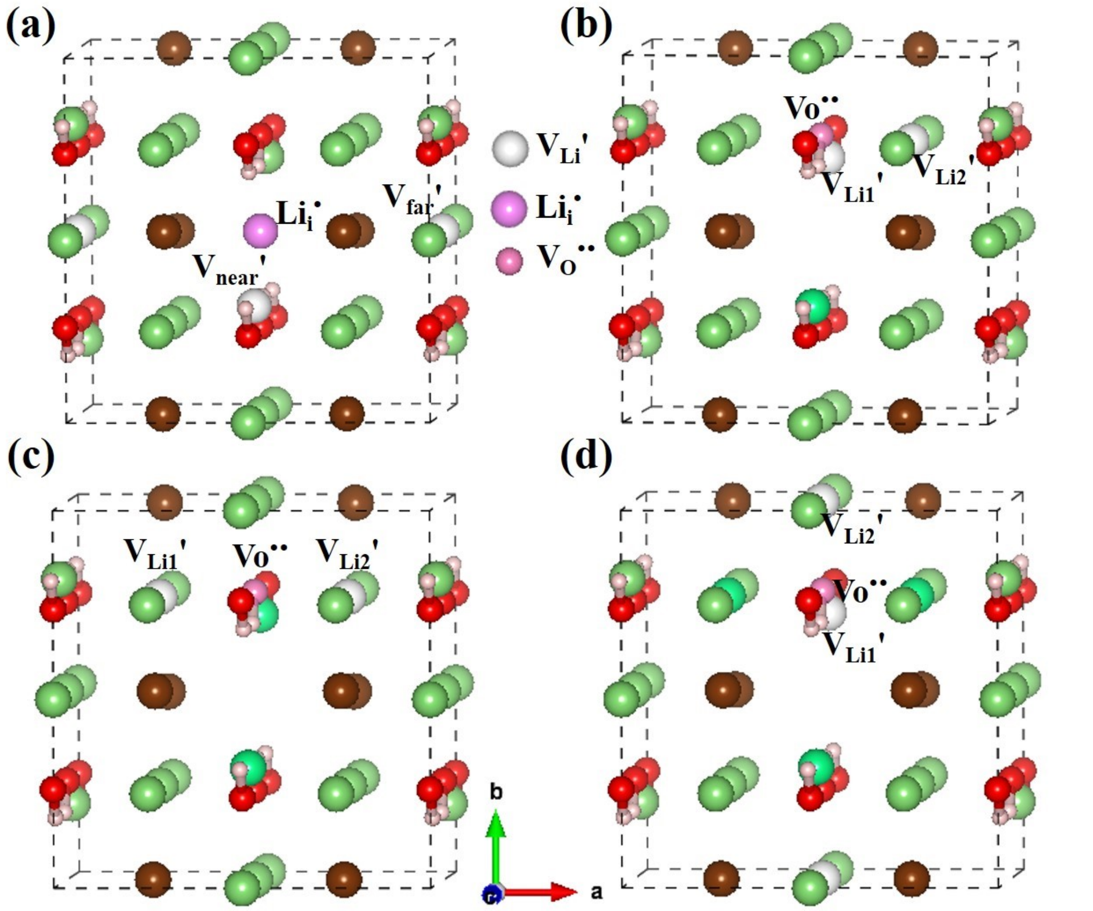

Figure S2. Possible defect configurations of the Li_2OHBr in (a) Frenkel defect pair ($V_{Li}^{'}$ - $Li_i^{\bullet}$). (b)-(d) show the adjacent, separated-1 and separated-2 Li_2O Schottky defect pair ($2V_{Li}^{'} - V_O^{\bullet\bullet}$),
